# Crosslinking Dependence of Direct Current Breakdown Performance for XLPE-PS Composites at Different Temperatures

**DOI:** 10.3390/polym13020219

**Published:** 2021-01-10

**Authors:** Liang Cao, Lisheng Zhong, Yinge Li, Jinghui Gao, George Chen

**Affiliations:** 1 State Key Laboratory of Electrical Insulation and Power Equipment, Xi’an Jiaotong University, Xi’an 710049, China; ge_chrissy@163.com (Y.L.); gaojinghui@mail.xjtu.edu.cn (J.G.); gc@ecs.soton.ac.uk (G.C.); 2 College of Engineering and Technology, Southwest University, Chongqing 400715, China

**Keywords:** crosslinked polyethylene, composite, breakdown strength, temperature dependence

## Abstract

In this paper, crosslinked polyethylene-polystyrene (XLPE-PS) composites with different degrees of crosslinking were fabricated by using different crosslinking agent contents and their direct current (DC) breakdown performance at 30~90 °C was investigated. Results show that with the increase of the degree of crosslinking, the crystallinity of XLPE-PS composites decreases gradually, but their DC breakdown strength demonstrates an increasing trend at 30~90 °C and the enhancement also increases with the rise of temperature. And as the degree of crosslinking increases, the elastic modulus of XLPE-PS composites is reduced and the loss tangent peak temperature decreases but the peak shifts to a lower value, which reveals the suppression of the relaxation process for crystallites. It is believed that high DC breakdown strength with good temperature stability for XLPE-PS composites with a larger degree of crosslinking is attributable to the presence of PS and suppression in the formation of crystallites due to crosslinking.

## 1. Introduction

Crosslinked polyethylene (XLPE) has long been favored as an insulating material for extruded high-voltage (HV) alternating-current (AC) power cable systems due to its benefits of outstanding electrical, mechanical and thermal properties [1], but in the case of direct-current (DC), with power cable systems trending towards higher voltages and a larger capacity, one of the urgently desired characteristics for polyethylene insulating materials is a high DC breakdown strength with good temperature stability [2]. In the past decades, various methods have been developed to enhance the DC breakdown strength of polyethylene, and nanocomposites have arisen much attention in this context. Nanofillers like MgO, Al_2_O_3_, SiO_2_, TiO_2_ and so on can improve the DC breakdown strength of polyethylene [3,4,5,6]. Based on lots of positive results acquired, nanocomposite addition is considered as one of the most promising techniques to realize the target of improving DC breakdown strength as well as related good temperature stability. However, it has been reported that the effect of adding nanofillers into polyethylene or XLPE on DC breakdown strength is inconsistent, and decreased DC breakdown strength of nanocomposites is even observed, which may come from the diversity of sample process or moisture absorption by the nanofillers [7,8]. Moreover, it is also of concern that internal stresses due to the mismatch of thermal expansion coefficients might lead to the insulation failure when a high electric field is applied [9].

To develop polyethylene-based materials with more stable DC breakdown behaviors, our previous study proposed a method of deliberately introducing polystyrene (PS) to enhance the high-temperature DC dielectric performance of XLPE and it is considered that the enhancement of DC dielectric performance may mainly rely on the formation of pinning structures, in which the crosslinking process plays a vitally important role [10]. For pure XLPE, the crosslinking process can change the morphology, thus affecting the DC breakdown strength. It has been reported that an increased degree of crosslinking would lead to a reduced breakdown strength for XLPE [11]. However, for XLPE-PS composites, the effect of different degrees of crosslinking on the DC dielectric performance is a consideration for the practical application in DC cable insulation and remains unknown, which needs a further investigation.

In this study, the effect of crosslinking on the morphology and DC breakdown performance of XLPE-PS composites was investigated. XLPE-PS composites with different degrees of crosslinking were prepared with different crosslinking agent contents. Their DC breakdown properties at 30~90 °C, space charge characteristics and morphology features such as gel content, crystallinity and thermal mechanical performance, were analyzed. It was notably observed that the degree of crosslinking has a positive influence on the DC breakdown strengths of XLPE-PS composites in the temperature range of 30~90 °C.

## 2. Materials and Methods

### 2.1. Sample Preparation

The matrix resin used for XLPE-PS composites was a commercial LDPE with a density of 0.92 g/cm^3^ (23 °C), a melt index of 2.0 g/10 min (190 °C, 2.16 kg), and an amorphous PS with a density of 1.05 g/cm^3^ (23 °C) and a melt index of 0.3 g/10 min (190 °C, 2.16 kg) was introduced by blending. Dicumyl peroxide (DCP) was used as the crosslinking agent and antioxidant 300 is selected.

Firstly, pellets of LDPE and PS as well as antioxidant were mixed using a twin-screw extruder at 170 °C with a rotation speed of 60 r/min in which only 1 part per hundred of resin (phr) of PS was employed [10]. Then a series of DCP contents including 0.8, 1, 1.3 and 2 phr were added into pellets of the blends by soaking at 70 °C for 24 h. Finally, samples with different dimensions were obtained by hot-pressing. After molding at 120 °C and 15 MPa for 5 min, plate samples were treated at 180 °C and 15 MPa for 15 min to complete the crosslinking process, and then cooled to room temperature. For the convenience of description, the designations as listed in Table 1 was used to represent the composites with different DCP contents. Before measurements, all samples were pre-treated in a vacuum oven at 70 °C for 24 h to remove crosslinking byproducts and release internal stress [12,13].

### 2.2. DC Dielectric Performance Measurements

DC breakdown tests were performed on a pair of cylindrical stainless-steel electrodes with a diameter of 25 mm. The electrodes were immersed in vegetable transformer oil to avoid surface flashover. The test temperature was maintained at 30, 50, 70 and 90 °C, respectively. DC voltage with a ramping rate of 1 kV/s was applied to the samples with a thickness of approximately 250 μm. For each recipe at a certain temperature, breakdown tests were repeated for 20 times and DC breakdown strength was acquired by the applied voltage divided by the thickness. The two-parameter Weibull distribution was used to analyze the results as follows [11,14,15]:

*P*_f_(*E*) = 1 − exp[ − (*E*/*E*_0_)*^β^*] (1)

where *P*_f_(*E*) is the cumulative failure probability in %, *E* is the applied electric field strength in kV·mm^−1^, *E*_0_ is the scale parameter, which represents the breakdown strength when the cumulative failure probability equals to 63.2% in kV·mm^−1^, and *β* is the shape parameter, which indicates the dispersion of acquired results. In this study, *E*_0_ is employed to characterize the DC breakdown strength of the composites.

The cumulative failure probability could be calculated by [16,17]:

*P*_f_(*E_i_*) = (*i* − 0.44)/(*n* + 0.25) (2)

where *P*_f_(*E_i_*) is the cumulative failure probability corresponding to the electric filed strength *E_i_* in %, *i* is the number of applied electric field strength arranged in the increment sequence, *n* is the total number of breakdown tests conducted under the same conditions (*n* equals 20 in this study).

The pulsed electro-acoustic (PEA) method was used to evaluate space charge behaviors in composites. The upper electrode is semi-conductive and the lower electrode is made of aluminum. Electric field strengths including 20 kV/mm and 40 kV/mm were applied to the samples with a thickness of about 250 μm. Silicon oil was used as acoustic coupling agent [18,19]. All the measurements lasted 60 min and were performed at room temperature.

### 2.3. Structural Characterization

In order to verify the condition of PS as the dispersed phase in polyethylene, cross sections of cryogenically fractured samples were observed with a scanning electron microscope (SEM). Samples were first immersed in liquid nitrogen for 3 min and then fractured in air immediately. Coated with thin gold layers, the morphologies of samples were acquired by SEM. 

The crosslinking degree of samples was assessed by gel content [11,20]. Each sample was cut into small cubes with a dimension of about 0.5 mm × 0.5 mm × 0.5 mm. A 120-mesh stainless-steel bag with 0.300 ± 0.015 g cubes was immersed into xylene at 110 °C for 24 h, then the bag was dried to a constant weight at 150 °C. Gel content was calculated as the percentage ratio of the final weight to the initial weight for samples. Gel content measurement for each recipe was repeated for 3 times. 

Differential scanning calorimetry (DSC) method was adopted to investigate melting and crystallization behaviors of composites. Samples of about 5.0 ± 0.5 mg were put into aluminum pans and measurements were performed at a nitrogen atmosphere. These samples were firstly heated from 30 to 200 °C at a rate of 10 min/°C, and hold for 3 min at 200 °C, then cooled to 30 °C with a rate of 10 min/°C. The crystallinities of samples could be calculated according to the following equation:

*χ*_c_ = Δ*H*_m_/Δ*H* × 100% (3)

where *χ*_c_ is the calculated crystallinity in %, Δ*H*_m_ is the measured melting enthalpy of composite in J·g^−1^, Δ*H* is melting enthalpy for 100% crystalline LDPE (287.3 J·g^−1^) [21].

### 2.4. Dynamic Mechanical Analysis

A dynamic mechanical thermal analyzer was utilized to study thermo-mechanical properties of composites, which may reveal the motion of molecule chains and difference of internal structure in the test temperature range. Double cantilever mode was employed and samples with a size of 60 mm × 10 mm × 3 mm were measured with a frequency of 1 Hz in the temperature range of 10~100 °C at a heating rate of 3 °C/min.

## 3. Results

### 3.1. DC Breakdown Properties

**Figure 1:**
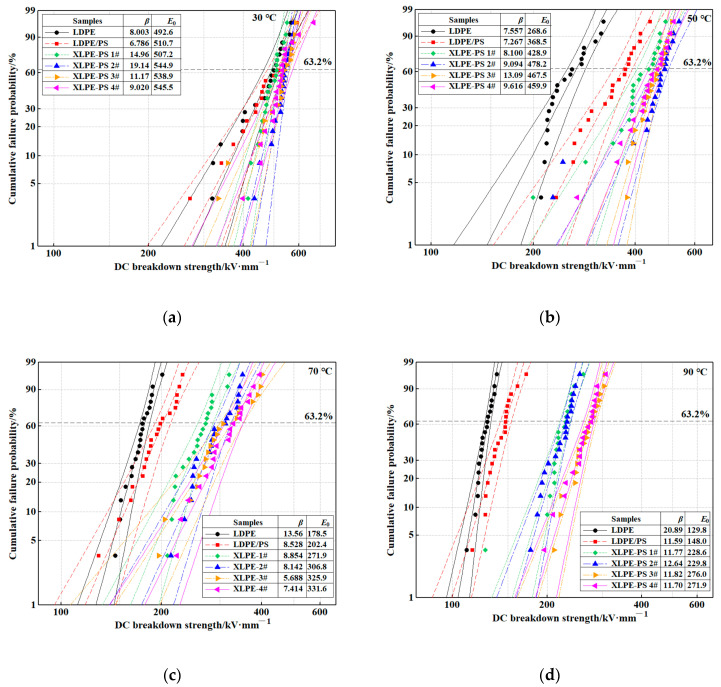
Weibull distribution of DC breakdown strength for samples at: (**a**) 30 °C; (**b**) 50 °C; (**c**) 70 °C; (**d**) 90 °C.

Figure 1 presents the Weibull distribution of DC breakdown strength for samples at 30~90 °C. Despite the phenomenon that DC breakdown strengths of all samples tend to decrease with the increase of temperature, but it could be noticed that compared with pure LDPE, DC breakdown strengths of composites are enhanced from 30 to 90 °C. The enhancement of DC breakdown strength differs in the composites with different DCP contents, which is quite different from pure XLPEs as shown in Figure S1 of the Supplementary Materials and results from Yan et al. [11].

**Figure 2:**
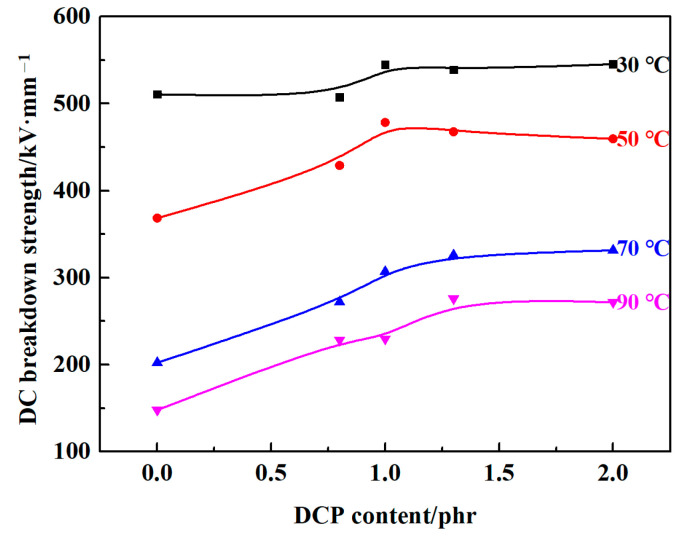
DCP content dependence of DC breakdown strength for samples at 30, 50, 70 and 90 °C.

Figure 2 describes effect of DCP content on DC breakdown strength in composites at 30, 50, 70 and 90 °C in detail. With the increase of DCP content, DC breakdown strengths of composites tend to increase in the test temperature range. Compared with LDPE/PS, DC breakdown strengths of XLPE-PS 4# are increased by 6.8% at 30 °C, 19.9% at 50 °C, 63.8% at 70 °C, and 83.7% at 90 °C respectively, which indicates that the rate of enhancement increases as the temperature rises.

**Figure 3:**
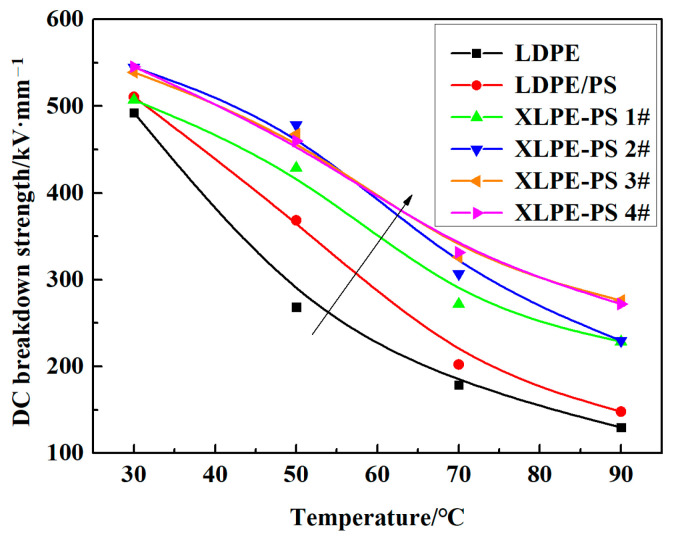
Temperature dependence of DC breakdown strength for samples.

Furthermore, the temperature dependence of DC breakdown strength for composites was compared, as shown in Figure 3. It can be seen that with the increase of temperature, the DC breakdown strength of each composite has a quite different descending trend. Notably, with the increase of DCP content, the temperature dependence of DC breakdown strength for composite become weak gradually. The temperature dependence of DC breakdown strength for XLPE-PS 3# and XLPE-PS 4# has little difference, and both of them show the weakest dependence on temperature.

### 3.2. Space Charge Behaviors

**Figure 4:**
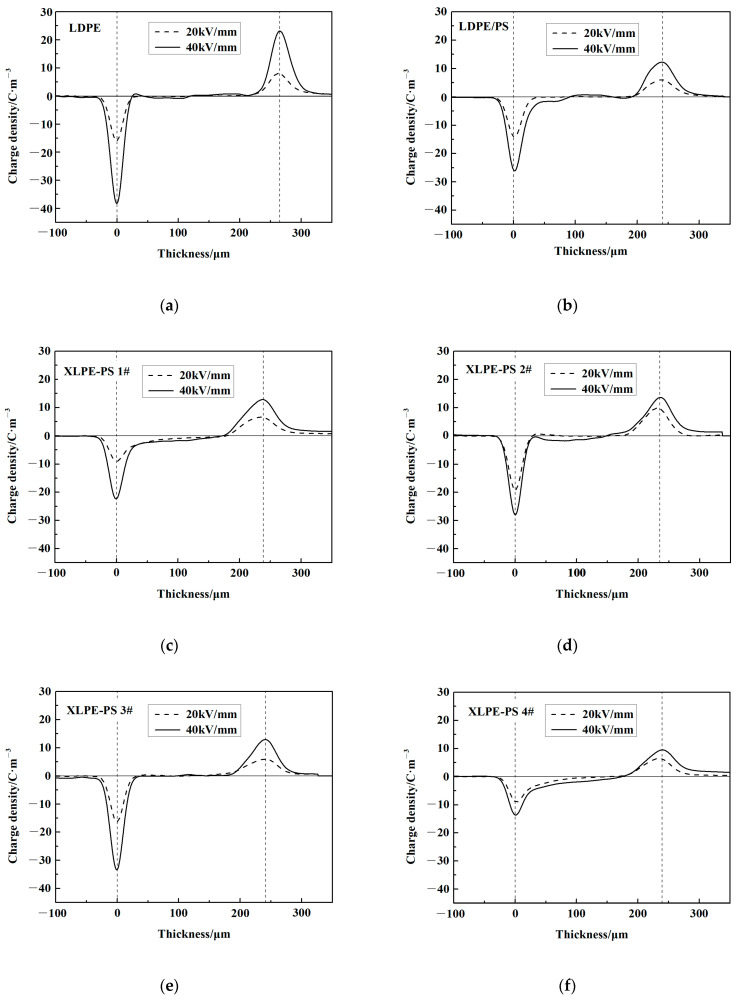
Space charge distribution in: (**a**) LDPE; (**b**) LDPE/PS; (**c**) XLPE-PS 1#; (**d**) XLPE-PS 2#; (**e**) XLPE-PS 3#; (**f**) XLPE-PS 4#.

Space charge behavior of polymeric insulation for cables is one of essential factors that prevent polymers from application in DC situations. Space charge accumulation in insulating materials can result in a distortion of the electric field and even failure. Figure 4 shows the space charge distribution in samples under 20 kV/mm and 40 kV/mm. For pure LDPE as shown in the Figure 4a, there is no obvious space charge accumulation under 20 kV/mm, but a small amount of heterocharge could be observed near the cathode under 40 kV/mm. It is considered that this heterocharge comes from ionization of impurities or transport of injected charges [18,22,23]. For composites, there is also no obvious space charge accumulation under 20 kV/mm in the composites except for XLPE-PS 1# and XLPE-PS 2# as shown in Figure 4c,f, where a negative charge accumulates near the cathode as homocharge and could be noticed almost across the whole samples. It is believed that homocharge comes from injected charges which are captured by localized states near the electrode. When the applied electric field increased to 40 kV/mm, apparent negative charge accumulates in the composites except for XLPE-PS 3#. In crosslinked samples with space charge accumulated as shown in Figure 4c,d,f, the areas of accumulated negative charge are larger than that in LDPE/PS. Notably, XLPE-PS 3# shows the least space charge accumulation among all composites, which helps to decrease the degradation of composites.

### 3.3. Cryogenically Fractured Morphology

**Figure 5:**
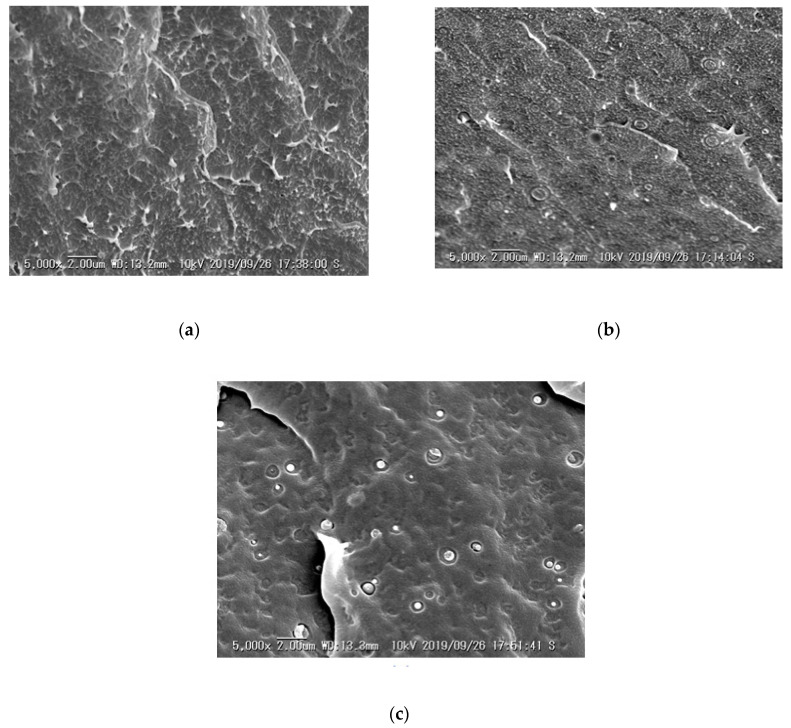
SEM images of cross sections for cryogenically fractured samples: (**a**) LDPE; (**b**) LDPE/PS; (**c**) XLPE-PS 3#.

The SEM images of cross sections for fractured samples LDPE, LDPE/PS and XLPE-PS 3# are shown in Figure 5. There is only a slight difference in XLPE-PS composites, thus just XLPE-PS 3# is presented. As expected, PS and polyethylene show a phase separation, and PS disperses in the matrix as spherical particles with a size ranging from nanometers to micrometers shown in Figure 5b,c. However, obvious differences could be observed among LDPE, LDPE/PS and XLPE-PS 3#. Comparing LDPE and LDPE/PS, it could be noted that the bulge generated by fracture become dense with the introduction of PS, which might be attributed to changes in the size of spherulites as well as the conditions of the amorphous region. 

As for LDPE/PS and XLPE-PS 3#, differences in the matrix and PS particles could be observed. Bulges in the matrix caused by fracture disappear or cannot be distinguished in crosslinked samples, which reveals that crosslinking process enhances the interactions between that molecular chains for polyethylene. Instead of cavities in LDPE/PS as shown in Figure 5b, PS particles in XLPE-PS 3# crack along the cross section, which has been ascribed to the crosslinking between PS and polyethylene [10].

### 3.4. Degree of Crosslinking and Crystallinity

**Figure 6:**
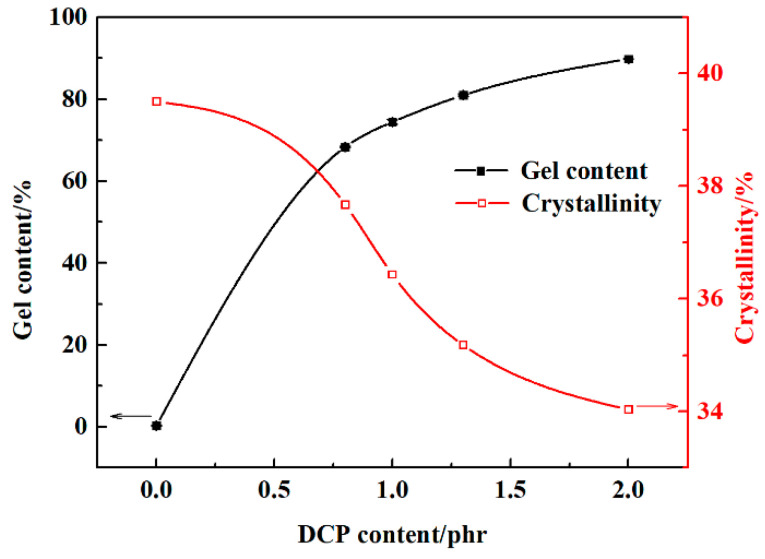
DCP content dependence of gel content and crystallinity for samples.

As shown in Figure 6, without crosslinking the gel content of LDPE/PS is approximately 0. With the increase of DCP content, the gel content of composites tends to increase and the growth rate of gel contents decreases gradually, which shows a trend to saturate. When the DCP content is 2 phr, namely in XLPE-PS 4#, the gel content reaches 89.8%. 

Correspondingly, the crystallinities of composites decrease gradually with the increase of DCP content. The crystallinity of LDPE/PS is about 39.5%, but when the DCP content reaches 2 phr, the crystallinity of XLPE-PS 4# is decreased to 34.0%. The DCP content dependence of the degree of crosslinking and crystallinity for composites are similar to pure XLPE (referring to Figure S2 in the Supplementary Materials) as well as the results of Yan et al. [11]. This may be attributed to the formation of crosslinking nodes in crosslinked samples. The existence of a crosslinking network limits the motion of the molecular chains and prevents them from forming a periodical structure. Thus, the increased degree of crosslinking leads to a decreased crystallinity in XLPE-PS composites.

**Figure 7:**
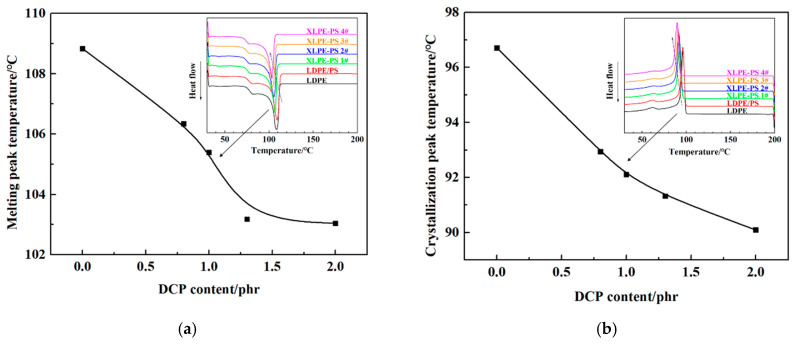
Effects of DCP content on melting and crystallization behaviors of samples: (**a**) melting process; (**b**) crystallization process.

Moreover, as shown in Figure 7a, the melting peak temperature decreases gradually with the increase of DCP content. According to the Thomson-Gibbs equation, the thickness of lamellae for polyethylene can be calculated as follows:

*L* = 2*σT*_m_^0^/(Δ*H*_v_*(*T*_m_^0^ − *T*_m_)) (4)

where *L* is the thickness of lamella in nm, *σ* is the lamellar surface free energy (0.07 J·m^−2^), *T* 0 m is equilibrium melting point of polyethylene (414.5 K), and Δ*H*_v_ is the melting enthalpy of lamella with infinite thickness for polyethylene (2.88 × 10^8^ J·m^−3^) [21,24], and *T*_m_ is the measured melting peak point of samples in K. Therefore, a lower melting peak temperature corresponds to a thinner lamella, and the decrease of melting peak temperature with the increase of DCP content indicates that lamellae of composites tend to decrease.

As for crystallization process of composites, two peaks could be observed, shown in Figure 7b. A main crystallization peak appears in the temperature range of 90~100 °C, and a secondary crystallization peak appears at about 63 °C. It is interesting to notice that with the increase of DCP content the main crystallization peak temperature descends gradually, while the secondary-crystallization peak temperature remains at almost the same value.

### 3.5. Thermo-Mechanical Performance

**Figure 8:**
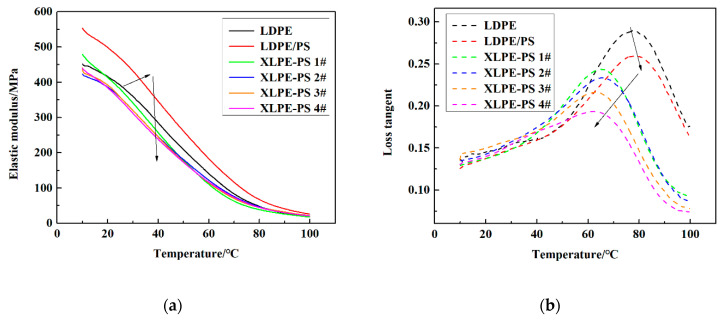
Dynamic mechanical behaviors of samples with different DCP contents: (**a**) elastic modulus; (**b**) loss tangent.

The thermo-mechanical behaviors of samples are shown in Figure 8. As can be seen that with the increase of temperature, the elastic modulus of all samples tends to decease. This phenomenon may result from an enhancement of segment or chain movement activated by the temperature increment [8]. Compared with pure LDPE, the addition of 1 phr PS increases the elastic modulus in the test temperature range of 10~100 °C, but after crosslinking, the elastic modulus of composites is decreased and with the increase of DCP content, the elastic modulus of composites shows a decreasing trend. 

Meanwhile, there is only one loss tangent peak appearing in the temperature range of 10~100 °C for each sample, which has been attributed to the melting process of crystallites for polyethylene [25]. Compared with pure LDPE, the peak of loss tangent for LDPE/PS shifts to a higher temperature and the corresponding peak value of loss tangent is lower. However, in crosslinked composites, with the increase of DCP content, the peak value of loss tangent for crosslinked samples tends to decrease but the peak of loss tangent shifts to a lower temperature.

## 4. Discussion

Semi-crystalline polyethylene possesses a morphology of spherulites formed by ordered lamellae and amorphous phases between these lamellae. Crystallinity is known as an important factor in determining the breakdown strength of polyethylene and the results from Niwa, Wang and Liu et al. show that the increase of crystallinity for polyethylene may enhance its breakdown strength [26,27,28]. By combining the results in Figure 2 and Figure 6, composites with a lower crystallinity and a thinner lamella show a higher DC breakdown strength, especially at 70 °C and 90 °C. Therefore, it could be inferred that crystallinity may not be the predominant factor that changes the DC breakdown behaviors of XLPE-PS composites. In contrast, it is reasonable to believe that the condition of amorphous area plays a vitally important role in improving DC breakdown strength for XLPE-PS composites. Crosslinking can change the microstructure of polyethylene, especially in the presence of PS. In the amorphous area, there exist impurities, crosslinking points and cavities. Due to the incompatibility of PS and polyethylene, PS would be located in the amorphous area [10]. The role of dispersed PS particles in XLPE-PS composites may be embodied in two aspects. On one hand, the existence of benzene rings could absorb the energy of high-energy electrons, reducing the risk of breakdown [29]. On the other hand, the crosslinking between PS and polyethylene improves their compatibility, leading to the decrease of defects in the interfacial area of PS and polyethylene. However, as presented in Figure 4, composites with different DCP contents show different space charge accumulation results, which makes local electric field distorted and reduces the DC breakdown strength. Therefore, there is a slight difference in the trend in crosslinking dependence of DC breakdown behavior for composites at different temperatures. 

The temperature dependence of DC breakdown strength for composites with different DCP content may be related to microstructure changes induced by temperature. It has been commonly thought that the crystalline area has a higher breakdown strength than the amorphous area does. With the rise of temperature, crystallites from secondary crystallization would turn into amorphous area, leading to the enlargement of the amorphous area, which contributes to the decreased DC breakdown strength of polyethylene at elevated temperatures. However, combining effects of DCP content on the secondary-crystallization peak in Figure 7b and shifts of loss tangent peak in Figure 8b, the increase of degree of crosslinking suppresses the formation of crystallites and reduces their content, which makes the ratio of amorphous area relatively reduced. Thus, the DC breakdown strength of crosslinked composites at 70 °C and 90 °C is enhanced and the temperature dependence of DC breakdown strength becomes weak.

## 5. Conclusions

The effects of the degree of crosslinking on the DC breakdown behaviors of XLPE-PS composites at 30~90 °C were investigated in this study. With the introduction of PS into polyethylene, the crosslinking process remarkably enhances the DC breakdown strength of polyethylene, and with the increase of degree of crosslinking, the DC breakdown strength of composites shows an increasing trend, the enhancement of which becomes larger at 70 °C and 90 °C. The related structures show that crosslinking has the role of weakening the temperature dependence of DC breakdown strength for composites related to the presence of PS in the amorphous area and suppression of the formation of crystallites for polyethylene by crosslinking.

## Figures and Tables

**Table 1:** Composites prepared in this study.

Designation	LDPE Content/phr	PS Content/phr	DCP Content/phr
LDPE	100	0	0
LDPE/PS	100	1	0
XLPE-PS 1#	100	1	0.8
XLPE-PS 2#	100	1	1
XLPE-PS 3#	100	1	1.3
XLPE-PS 4#	100	1	2

## Data Availability

Data is contained within the article and supplementary material.

## Supplementary Materials

The following are available online at https://www.mdpi.com/2073-4360/13/2/219/s1, Table S1: prepared XLPEs with different crosslinking degrees, Figure S1: Weibull distribution of DC breakdown strength for LDPE and XLPEs at: (a) 30 °C; (b) 50 °C; (c) 70 °C; (d) 90 °C, Figure S2: DCP content dependence of gel content and crystallinity for XLPEs.
