# Supplementary material for: Crosslinking Dependence of Direct Current Breakdown Performance for XLPE-PS Composites at Different Temperatures
Source: Polymers (Basel). 2021 Jan 10;13(2):219. doi: 10.3390/polym13020219 (PMC7827150; doi:10.3390/polym13020219)
Supplement: Supplementary file 1 [file polymers-13-00219-s001.pdf]

Supplementary Materials

The fabrication process of XLPEs listed in Table S1 below was similar to XLPE-PS composites, and the only difference is with PS or not.

Table S1. prepared XLPEs with different crosslinking degrees.

Designation	LDPE Content/phr	DCP Content/phr
LDPE	100	0
XLPE 1#	100	0.8
XLPE 2#	100	1
XLPE 3#	100	1.3
XLPE 4#	100	2

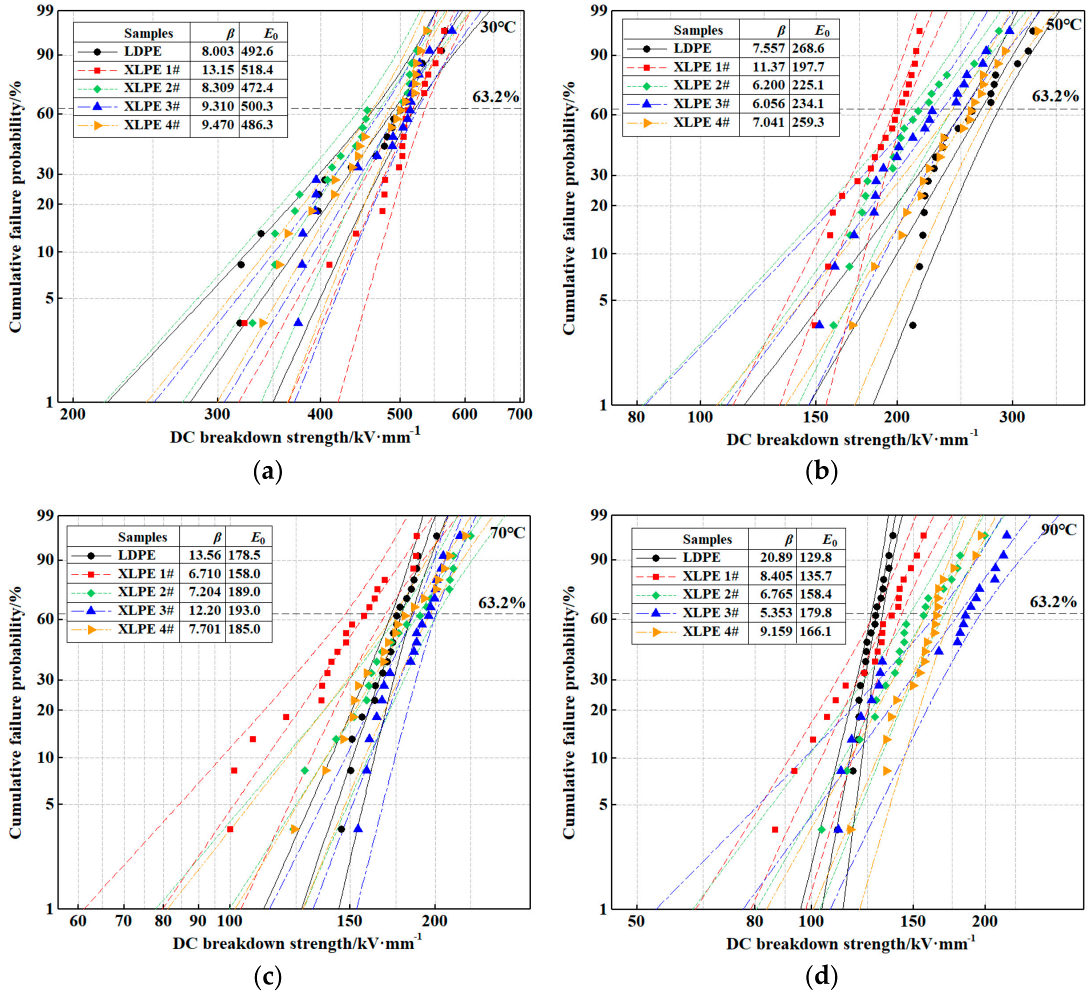

Figure S1. Weibull distribution of DC breakdown strength for LDPE and XLPEs at: (a) 30°C; (b) 50°C; (c) 70°C; (d) 90°C.

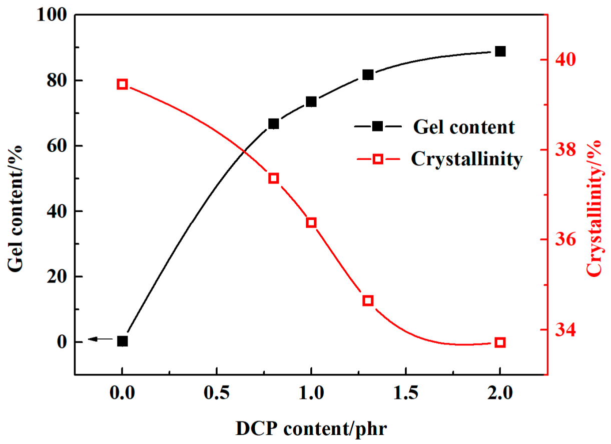

Figure S2. DCP content dependence of gel content and crystallinity for XLPEs.
